# Synthesis of the clinical utilities and issues of intraoperative imaging devices in clinical reports: a systematic review and thematic synthesis

**DOI:** 10.1186/s12911-025-02915-x

**Published:** 2025-02-10

**Authors:** Hiroyuki Suzuki, Yusuke Tsuboko, Manabu Tamura, Ken Masamune, Kiotaka Iwasaki

**Affiliations:** 1 Cooperative Major in Advanced Biomedical Sciences, Joint Graduate School of Tokyo Women’s Medical University and Waseda University, Tokyo, 162-8480 Japan; 2 Sony Computer Science Laboratories, Inc., Tokyo Research, Tokyo, 141-0022 Japan; 3 Waseda Research Institute for Science and Engineering, Waseda University, Tokyo, 162-8480 Japan; 4 Institute for Medical Regulatory Science, Comprehensive Research Organization, Waseda University, Tokyo, 162-8480 Japan; 5 Tokyo Women’s Medical University, Faculty of Advanced Techno-Surgery, Tokyo, 162-8480 Japan; 6 Department of Modern Mechanical Engineering, School of Creative Science and Engineering, Waseda University, Tokyo, 169-8555 Japan; 7 Department of Integrative Bioscience and Biomedical Engineering, Graduate School of Advanced Science and Engineering, Waseda University, Tokyo, 162-8480 Japan

**Keywords:** Qualitative evidence synthesis, Thematic analysis, Intraoperative imaging devices, Intraoperative optical coherence tomography

## Abstract

**Background:**

Intraoperative imaging devices (i-ID), such as intraoperative optical coherence tomography (iOCT), offer surgeons critical insights previously unobservable, enhancing surgical precision and safety. Despite their benefits, i-IDs present challenges that necessitate early identification and synthesis of clinical issues to promote safer surgical implementation. This study aims to explore the potential of Qualitative Evidence Synthesis (QES) for synthesising qualitative evidence from clinical reports regarding the clinical utility and issues associated with iOCT devices.

**Methods:**

In June 2022, we conducted a systematic literature search using PubMed, Web of Science, Embase, and the Cochrane Library for articles on iOCT for retinal surgery. Criteria included articles in English, with at least ten cases, and providing qualitative insights into iOCT’s utilities and issues. We performed thematic synthesis from the identified articles using qualitative data analysis software, beginning with initial coding of the ‘Results’ and ‘Discussion’ sections to create themes reflecting iOCT’s utilities and issues. The created themes were further refined through axial coding and were used to construct a model illustrating iOCT’s potential influence on patient outcomes. The reliability and validity of the themes were ensured through independent coding, expert consultations, and iterative revisions to achieve consensus among reviewers.

**Results:**

The QES approach enabled systematic data extraction and synthesis, providing a comprehensive view of both the utilities and issues associated with iOCT. Our findings emphasise the significant role of iOCT in enhancing decision-making, specifically in membrane peeling tasks and in detecting preoperatively undetected conditions such as full-thickness macular holes. This study also revealed critical insights into the technical challenges associated with iOCT, including device malfunctions and procedural interruptions, which are vital for improving device safety and integration into surgical practice.

**Conclusion:**

The application of QES facilitated a thorough investigation into the clinical utilities and issues of iOCT, encouraging the application of this method in the ongoing evaluation of i-ID technologies. This initial experience with QES confirms its potential in synthesising qualitative clinical data and suggests its applicability to other i-ID modalities. This approach enhances the reliability of findings and provides a solid foundation for assessing clinical utilities and issues for policymakers and medical specialists.

**Supplementary Information:**

The online version contains supplementary material available at 10.1186/s12911-025-02915-x.

## Background

Intraoperative imaging devices (i-ID) equipped with innovative technologies are anticipated to enhance the efficacy and safety of surgical treatments. For instance, surgeons can now observe retinal microsections using intraoperative optical coherence tomography (iOCT) and visualise the brain and organs with intraoperative magnetic resonance imaging (iMRI) and intraoperative computed tomography (iCT), which were previously unobservable during surgeries. Intraoperative decision-making, facilitated by the crucial additional information provided by i-ID, potentially leads to more precise surgical manoeuvres and improved patient outcomes. However, it is common for new medical modalities to coexist with both advantages and disadvantages. Therefore, in addition to assessing the clinical utilities of the i-ID, synthesising and identifying clinical issues early on is crucial to promote their implementation in surgical rooms and enhance the safety of these medical devices.

When synthesising clinical evidence regarding innovative medical devices, including i-ID, specific challenges arise that are distinct from those encountered with pharmaceuticals. Firstly, during the early market introduction phase, the safety and efficacy of these devices are influenced by human factors such as learning curves and human errors. These factors impede randomised controlled trials and lead to less uniform clinical report descriptions [1]. Secondly, the advantages of i-ID often emerge from intraoperative adjustments in surgical behaviour, which are driven by the use of intraoperative images. For example, clinical trial endpoints for iOCT may include “alterations of intraoperative decision-making”, dependent on the surgeon’s experience [2, 3]. Thirdly, the high installation costs of i-ID hinder large-scale clinical trials, and the limited number of clinical reports makes statistical synthesis difficult. Given these issues, “How to synthesise heterogeneous qualitative descriptions of clinical reports in i-ID” remains a critical challenge in this field.

Various methods have been proposed for qualitative evidence synthesis (QES) in systematic reviews (SR), yet traditionally, the descriptions of qualitative research have been the primary datasets for synthesis [4]. SR with QES has primarily developed around qualitative research focusing on patient experiences in healthcare [5] and developers’ experiences in software engineering [6]. Although QES is still evolving, it has recently been formalised by the Cochrane Collaboration [7] and is also being used in policy-making [8, 9], making it an increasingly established method. Primary cases in QES are characterised by identifying and synthesising descriptive patterns from the dataset of multiple qualitative studies consisting of interviews or questionnaires. In contrast, clinical reports, such as case reports and clinical trials on medical devices, are generally focused on surgical procedures and clinical outcomes, which differ from the general datasets that QES traditionally targets. Therefore, it seems meaningful to explore the feasibility of QES for the descriptions in clinical reports to further assess potential applications of this methodology.

This study aims to report the initial experiences investigating the potential of QES for synthesising the clinical utility and issues of i-ID from clinical reports. We selected iOCT as a model case of emerging modalities in i-ID to analyse its clinical utilities and issues using a QES approach. As large-scale RCTs for iOCT have not yet been conducted, a comprehensive synthesis of heterogeneous descriptive data from clinical reports is valuable. We employed a QES approach—thematic synthesis [10]—to create themes representing the clinical utilities and issues of iOCT. Based on these themes, we constructed a model illustrating the influence of iOCT on patient outcomes.

## Methods

**Fig. 1:**
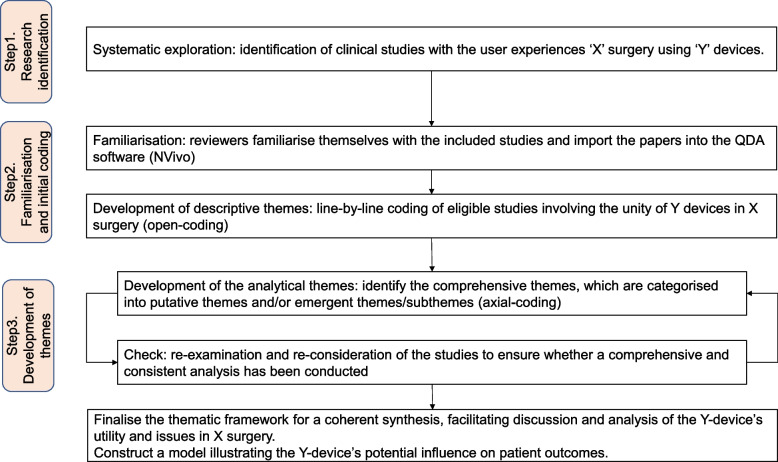
Steps of thematic evidence synthesis for intraoperative imaging devices. Step 1: Identification through systematic article search. This step was conducted using the PRISMA 2020 Flow Diagram to enable comprehensive exploration. The parameters shown in this figure (‘X’ and ‘Y’) define the target clinical application and intraoperative imaging modalities, respectively. In this study, we set ‘X’ to ‘Retinal’ and ‘Y’ to ‘iOCT’. Step 2: Familiarisation and initial coding. In this step, reviewers familiarised themselves with the included studies and imported the papers into QDA software. Line-by-line coding was performed, allowing each sentence to be indexed to a specific code. Step 3: Development of themes. This step involved a looping process. Initially, reviewers developed provisional themes to identify comprehensive themes and sub-themes. Subsequently, the reviewers examined the relationship between the developed codes and the provisional themes. If the provisional thematic framework was a coherent synthesis that facilitated discussion and analysis, the step was finalised; otherwise, Step 3 was repeated

Figure 1 presents a framework outlining the process from data acquisition of clinical reports to synthesising the clinical utilities and issues of i-ID from the qualitative descriptions, namely Qual-SR for i-ID. This framework was developed by utilising clinical reports as datasets to extract and synthesise qualitative evidence influencing patient outcomes through the approach of thematic synthesis [10]. The Qual-SR for i-ID consists of three key steps: 1) acquiring related clinical reports of i-ID through a systematic literature search, 2) synthesising qualitative evidence, and 3) building a model and mapping the developed themes from the clinical reports.

### Dataset acquisition

**Fig. 2:**
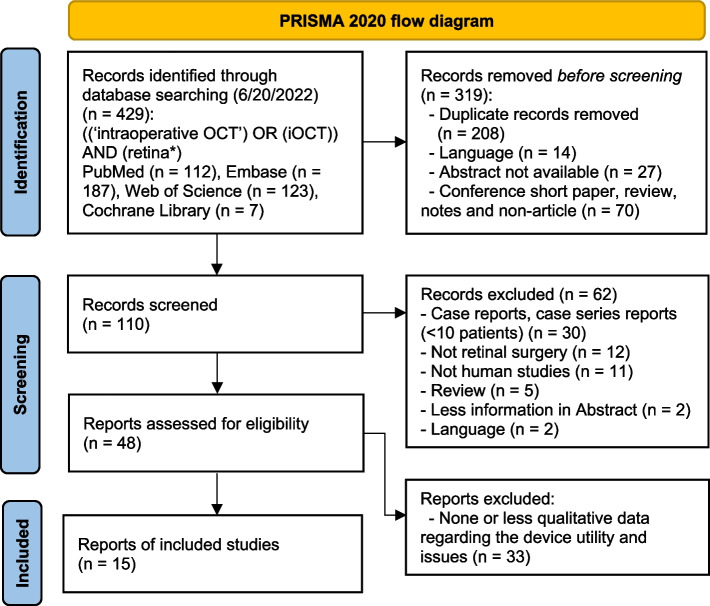
Systematic article exploration using the PRISMA 2020 Flow Diagram. Initially, to identify potential studies, we input the keywords ((‘intraoperative OCT’) OR (iOCT)) AND (retina*)) into four databases: PubMed, Embase, Web of Science, and the Cochrane Library. This search yielded 429 research candidates. Next, to screen these articles, we conducted checks for duplication, abstract availability, language, and article type. Furthermore, we screened each study according to the following criteria: 1) original articles, 2) written in English, 3) iOCT is the main research topic, 4) more than 10 cases, 5) clinical research, and 6) qualitative descriptions presenting the utility and/or technical issues of the iOCT. In total, 48 articles were screened based on title and abstract. Ultimately, 15 studies were deemed eligible for inclusion after a detailed eligibility assessment

The dataset acquisition process was conducted using the PRISMA 2020 Flow Diagram to enable comprehensive exploration. The checklist is provided in Supplemental Table 1. The parameters for the process of Qual-SR for i-ID shown in Fig. 1 were defined as ‘X’ = ‘Retinal’ and ‘Y’ = ‘iOCT’. Clinical reports related to iOCT for retinal surgery were searched in June 2022 by two independent investigators (H.S. and Y.T.) in the following databases: PubMed, Web of Science, Embase, and the Cochrane Library (Fig. 2). Search terms were chosen to cover descriptions of iOCT usage in retinal surgery: ((‘intraoperative OCT’) OR (iOCT)) AND (retina*). Eligible articles were identified through a three-step process: 1) an initial literature search, 2) screening of search results, and 3) eligibility evaluation of articles based on title and abstract. The main inclusion criteria were: 1) articles, 2) written in English, 3) iOCT as the main research topic, 4) cases > 10, 5) clinical research, and 6) qualitative description of utilities and issues. Abstracts were excluded if they were unavailable or lacked sufficient information for screening. Discrepancies in screening results were resolved through consultation.

### Synthesis of qualitative evidence using a QES method

This study employed thematic synthesis [10] among various QES methods due to its established application in medical research and its flexibility in synthesising qualitative data. Thematic synthesis, rooted in qualitative research, identifies and develops themes based on patterns observed in qualitative descriptions. The thematic development involved three steps: 1) familiarisation and initial coding, 2) axial coding, and 3) theme development. During initial coding, investigators familiarised themselves with the clinical reports using qualitative data analysis (QDA) software (NVivo, QSR International Inc., Burlington, MA, USA), manually creating categories line-by-line within the Results and Discussion sections of the reports. For example, sentence #1 was coded as “iOCT-assisted observation after membrane peeling, decided on additional peeling”. Sentence #2 was coded as “iOCT-assisted observation after membrane peeling, detected hole, and decided on gas tamponade”.

Example Sentence #1:

*Following peeling, 1/7 (14%) eyes with ERM were noted on iOCT to have a residual membrane within the macular arcade that the surgeon determined required additional membrane peeling due to its proximity to the fovea [figure].* ( [11], p.4).

Example Sentence #2:

*In one of these cases, after ERM and internal limiting membrane peeling, a small full-thickness hole inferior to the fovea was detected by SS-MIOCT through examination of 2D B-scans across the site of the hole [figure]. Decision was made to leave the eye filled with 20% SF6 as a tamponade agent to aid closure of the detected retinal break.* ([12], p.4).

Axial coding reorganised the initial codes by classifying and structuring them to express descriptions across individual studies. For instance, codes from examples #1 and #2 were synthesised into the revised code as “Identification of necessary additional surgical manoeuvres.”

In the theme development phase, the quality of the themes was enhanced by assessing the inter-rater reliability, involving coding by an independent reviewer. Firstly, the reviewer (H.S.) developed the tentative themes of utilities and clinical issues of i-ID from the datasets. These themes were compiled into a codebook that included definitions and example sentences. The codebook was refined through advice from multiple experts in medical devices (M.T., K.M., K.I.) during research meetings. Secondly, another reviewer (Y.T.) independently coded the same dataset using the refined codebook as a reference. Agreements and disagreements between the two reviewers’ coding results were analysed, and discussions were conducted to modify the themes until a consensus was reached.

### Model construction of the impact of iOCT on patient outcomes

Model construction aims to clarify the research questions (RQ) related to the subject of analysis and address these questions based on the developed themes, as reported in previous research [13]. The critical research question for iOCT involved the occurrence of desired or undesired intraoperative events during operations, which includes alterations in surgical decision-making or adverse events. Firstly, we defined the key components of the model as follows:

1. surgeons’ interventions
2. intraoperative OCT use
3. desirable/undesirable intraoperative events associated with iOCT use
4. patient outcomes

**Fig. 3:**
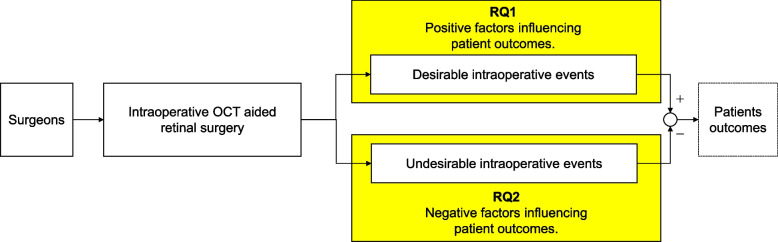
Research questions (RQs) and a tentative model illustrating the influence of iOCT on patient outcomes. The model comprises four elemental blocks: 1) intervention of surgeons, 2) iOCT-aided retinal surgery, 3) intraoperative events, and 4) patient outcomes. RQ1 investigates the positive factors in intraoperative events that influence patient outcomes, while RQ2 examines the negative factors arising from intraoperative events. Based on the identified themes, we developed appropriate responses to these RQs

Next, we framed factors influencing the desirable/undesirable intraoperative events as RQs. Figure 3 presents a tentative model illustrating iOCT's influence on patient outcomes. RQ1 addresses positive factors influencing patient outcomes, while RQ2 addresses negative factors. Appropriate responses to these RQs were formulated based on the identified themes.

### Mapping of the developed themes with the clinical reports

Visualising the developed themes and their sources of information is crucial for ensuring the transparency of the synthesis process. Using NVivo’s Matrix Coding Query, mapping was performed to determine the relevance of the themes to the literature.

### Quality appraisal

In June 2022, two investigators evaluated the level of evidence of the identified 15 research papers using the Oxford Centre for Evidence-Based Medicine 2011 Levels of Evidence (OCEBM Levels of Evidence) [14]. The OCEBM Levels of Evidence is a tool that evaluates the level of evidence in study designs using a five-tier scale. Meta-analyses of randomised controlled trials (RCTs) are often rated as level 1, RCTs as level 2, cohort studies as level 3, case series as level 4, and mechanism-based description as level 5. By answering seven questions related to the study design, the overall evidence level for each study was determined. Two independent researchers (H.S. and Y.T.) assessed the evidence level. In cases where their assessments differed, discussions were held to reach a consensus.

## Results

An initial search of databases identified 429 articles, of which 15 met the eligibility criteria for our analysis (Fig. 2). The quality of the studies was appraised at levels 3 ( [15, 16]) and 4 ( [11, 12, 17–27]).

Open and axial coding identified three main themes: 1) alternation of decision-making, 2) utility of the microsurgical procedure, and 3) troubles or surgical interruptions. These themes are further divided into several sub-themes (Table 1).

**Table 1:** Research mapping based on the developed themes of the clinical utilities and issues of iOCT in retinal surgery

Studies	i) Alternation of critical decision-making			ii) Utility of the microsurgical procedures		iii) Troubles or interruptions		
	1_Identification of necessary additional or alternative surgical manoeuvres	2_Prevention of unnecessary surgical manoeuvres	3_Identification of the preoperatively undetected lesions	1_Deep understanding of the microanatomy for the accurate execution of the surgical procedure or clear observation of the target lesions	2_Confirmation of completion of the surgical procedure	1_Device malfunction	2_Interruption of operation for image acquisition	3_Contamination
Abraham et al. [15]	*			*		*		
Ehlers et al. [11]	*	*	*	*	*			
Ehlers et al. [17]	*	*	*	*	*		*	
Ehlers et al. [18]	*	*			*		*	
Ehlers et al. [19]	*	*		*	*	*		*
Ehlers et al. [20]	*	*	*	*		*	*	
Ehlers et al. [21]	*	*						
Gabr et al. [12]	*	*		*				
Khan et al. [22]	*	*	*	*	*	*		
Kumar et al. [16]	*	*		*		*		*
Pfau et al. [23]	*			*			*	
Runkle et al. [24]	*	*	*		*	*		
Tao et al. [25]	*						*	
Yee et al. [26]	*	*				*	*	
Zakir et al. [27]	*			*	*			

*Indicates the applicability of each theme and sub-theme

### Theme 1: alternation of decision-making

Theme 1 encompasses changes in surgical decisions promoted by insights from i-ID. It includes three sub-themes: 1) identification of necessary additional or alternative surgical manoeuvres, 2) prevention of unnecessary surgical manoeuvres, and 3) identification of preoperatively undetected lesions.

#### Theme 1-sub.1: identification of necessary additional or alternative surgical manoeuvres

All 15 studies included descriptions categorised under this sub-theme [11, 12, 15–27]. This sub-theme involved altering decision-making to execute additional surgical manoeuvres owing to iOCT visualisation. In 12 studies, iOCT contributed to the determination of additional membrane peeling intraoperatively [11, 15–22, 24–26]. For example, surgeons believed that membrane peeling had been completed; however, iOCT revealed residual membranes. One study showed that surgeons detected a small full-thickness macular hole inferior to the fovea through an iOCT image after epiretinal membrane peeling [12], leading to the decision to use gas as a tamponade agent for closure.

#### Theme 1-sub.2: prevention of unnecessary surgical manoeuvres

Eleven of the 15 studies included descriptions categorised under this sub-theme [11, 12, 16–22, 24, 26]. This sub-theme demonstrated the device’s utility in reducing potential risk by eliminating unnecessary intraoperative procedures. Eight studies reported that iOCT helped prevent excessive membrane peeling with potential risks, such as with internal limiting membrane and epiretinal membrane [12, 17, 18, 20–22, 24, 26]. Three articles also described how iOCT prevented other potential risks [11, 16, 22]: one by enabling membrane peeling without using adjuncts such as indocyanine green [11]; another by preventing unnecessary and risky surgical manoeuvres, including laser or tamponade [22]; the third prevented a chorioretinal biopsy since the potential biopsy site was in a shallow layer of subretinal fluid [16].

#### Theme 1-sub.3: identification of the preoperatively undetected lesions

Five of the 15 studies included descriptions categorised under this sub-theme [11, 17, 20, 22, 24]. These studies described how iOCT is helpful for the intraoperative identification of preoperatively undetected lesions. For example, undetected full-thickness macular holes were identified intraoperatively due to the time lag between the preoperative scan in the clinic and the iOCT scan, promoting decisions for additional surgical tasks, including membrane peeling or gas tamponade [11].

### Theme 2: utility of the microsurgical procedure

Theme 2 highlights the utility of intraoperative imaging for precision in microsurgical procedures and is categorised into two sub-themes: 1) deep understanding of the microanatomy for the accurate execution of the surgical procedure or precise observation of the target lesions and 2) confirmation of completion of the surgical procedure.

#### Theme 2-sub.1: deep understanding of the microanatomy for the accurate execution of the surgical procedure or precise observation of the target lesions

Ten studies reported that iOCT enables surgeons to observe details that are typically difficult to visualise with conventional microscopes [11, 12, 15–17, 19, 20, 22, 23, 27]. For example, iOCT provides real-time views that help navigate through obscured surgical fields, such as those hindered by corneal oedema. It allows surgeons to see underlying membranes and other structures that are not visible to the naked eye, enhancing the precision of surgical interventions [19].

#### Theme 2-sub.2: confirmation of completion of the surgical procedure

Six studies describe the role of iOCT in confirming the completeness of procedures such as membrane peeling, which can be difficult under a conventional microscope due to the translucent nature of tissues. In these cases, surgeons often rely on staining with vital dyes for better visualisation. However, iOCT imaging allows confirmation of complete membrane removal without the need for additional dyes, thereby reducing the surgical risks associated with extra procedures [17, 18, 20, 23, 25, 26].

### Theme 3: troubles or surgical interruptions

Theme 3 includes reports of intraoperative troubles or interruptions associated with the use of iOCT, divided into three sub-themes: 1) device malfunction, 2) interruption of operation for image acquisition, and 3) contamination.

#### Theme 3-sub.1: device malfunction

Seven studies reported issues related to device malfunction, such as software-related problems, including imaging freezing, system reboots, and software errors, were identified in seven articles [15, 19, 20, 22, 24, 26]. In addition, hardware malfunctions, such as microscope failure and unresponsive foot pedals, were noted in four studies [16, 19, 20, 22].

#### Theme 3-sub.2: interruption of operation for image acquisition

Six of the 15 studies reported surgical interruption caused by iOCT scanning. The scanning time varied based on the iOCT modality used, ranging from 1.3 to 4.9 min. Examples include handheld OCT and microscope-mounted OCT systems [17, 18, 20, 23, 25, 26].

#### Theme 3-sub.3: contamination

Two of the 15 studies described incidents of contamination involving iOCT devices, surgical gloves, surgical instruments, and microscope handles [16, 19]. However, none of these contamination events directly affected the surgical field.

### Model construction of the impact of iOCT on patient outcomes

**Fig. 4:**
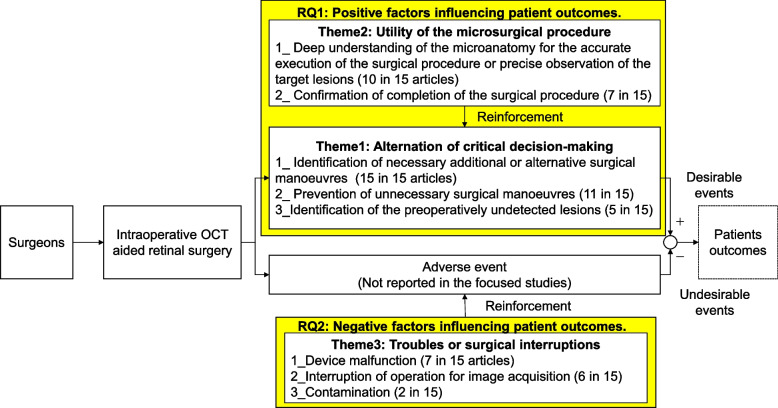
A model illustrating the influence of iOCT on patient outcomes. The model comprises seven blocks: 1) intervention of surgeons, 2) iOCT-aided retinal surgery, 3) the utility of the microsurgical procedure (Theme2), 4) troubles or surgical interruptions (Theme 3), 5) alternation of critical decision-making (Theme1), 6) adverse events, and 7) patient outcomes

A model was developed to describe the potential impact of iOCT on patient outcomes during retinal surgery, utilising the identified themes and sub-themes (Fig. 4). For RQ1 (positive factors influencing patient outcomes), we assigned Theme 1 (“Alternation of critical decision-making”) and Theme 2 (“Utility of the microsurgical procedure”), both reflecting the utility of iOCT. In contrast, for RQ2 (negative factors influencing patient outcomes), we assigned Theme 3 (“Troubles or surgical interruptions”), which highlights issues associated with the device.

The constructed model comprises seven components: 1) the intervention of surgeons, 2) the iOCT device, 3) the utility of the microsurgical procedure (Theme 2), 4) troubles or surgical interruptions (Theme 3), 5) alteration of critical decision-making (Theme 1), 6) adverse events, and 7) patient outcomes. This model illustrates that patient outcomes are potentially influenced by the intraoperative utility of iOCT in microsurgical procedures, which is closely linked to the alteration of crucial intraoperative decisions. Furthermore, troubles and interruptions associated with iOCT can lead to adverse events, thereby affecting patient outcomes.

### Mapping of the developed themes with the clinical reports

Table 1 presents the mapping of developed themes to the clinical studies, with asterisks (*) indicating which themes are described in each study. This mapping enhances transparency in evidence extraction, providing a clear understanding of the described utilities and issues in the clinical reports.

As shown in the table, the most frequently described utility of iOCT was the identification of necessary additional or alternative surgical manoeuvres (Theme 1–1), which was mentioned in all studies. In contrast, the most frequently described issue was device malfunction (Theme 3–1), reported in approximately half of the studies.

## Discussion

In this study, we investigated the feasibility of synthesising the clinical utilities and issues of i-ID using a QES method. The results of the QES for iOCT demonstrated its ability to comprehensively extract both clinical utilities and issues from clinical reports. Identifying such clinical issues is crucial for improving the safety of innovative medical devices such as i-ID, where dissemination and improvement occur concurrently after product launch [28]. The QES approach, involving line-by-line analysis of the entire target literature by independent reviewers, is inherently more comprehensive than single-reviewer data extraction commonly used in narrative reviews. Narrative synthesis, while expert-driven, is prone to selection bias [4, 6, 29, 30], potentially overlooking key utilities and issues described in clinical reports. Therefore, synthesising qualitative evidence using recognised QES methods is highly desirable.

Our case study of iOCT-assisted retinal surgery revealed qualitative evidence highlighting its clinical utilities. These include tasks such as membrane peeling (e.g., internal limiting membrane and epiretinal membrane) and in the intraoperative detection of preoperatively undetected diseases. Twelve of the 15 studies reported that residual membranes were revealed by iOCT-imaging after surgeons considered that their membrane peeling was completed, which contributed to determining additional surgical manoeuvres. Furthermore, eight of the 15 studies reported that the retinal cross-section provided by iOCT imaging helped confirm complete membrane removal, avoiding further unnecessary membrane peeling, whereas conventional microscopy did not provide a cross-sectional image of the microstructure during the operation. The reported recurrence rate of the epiretinal membrane due to residual membranes was relatively high, up to 20% [31]. This finding is notable in that iOCT-enabled alteration of intraoperative decision-making is connected with avoiding the potential risk of recurrence. In addition, five of the 15 studies reported that intraoperative identification of undetectable full-thickness macular holes by preoperative diagnosis resulted in an alteration of the surgical plan. Full-thickness macular hole, a common condition [32], can progress to larger holes and severe vision loss if left untreated [33]. These findings underscore how iOCT enhances surgical accuracy and completeness compared to conventional microscopy.

Importantly, the qualitative analysis also revealed clinical issues associated with iOCT devices in retinal surgery. Seven of the 15 studies reported surgical interruptions or image acquisition failures due to software malfunctions. Six studies described surgical interruptions caused by iOCT scanning time, with conventional handheld iOCT devices requiring approximately 5 min, compared to the approximately 1-min interruption time of more recent microscope-integrated iOCT systems launched in 2014. Two studies noted contamination risks of iOCT devices involving surgical devices or surgical gloves. Addressing such technical issues is critical, yet SRs on iOCT have rarely addressed these issues [34–36]. A key advantage of the QES approach proposed in this study is its ability to equally identify both utilities and technical issues.

Constructing a model of iOCT’s impact on patient outcomes in retinal surgery provides valuable insights into its intraoperative benefits and risks. The importance of model construction using qualitative evidence for future scenario predictions has been highlighted in fields such as sociology and pedagogy [37]. However, SRs in i-ID research have not traditionally focused on model construction. Aggregating and visualising descriptive clinical data into a model allows for informed discussions on evaluation criteria in clinical research. The comprehensive modelling presented here, which integrates both clinical utilities and issues, offers valuable insights for rapidly improving device efficacy and safety.

Our findings also inform the design of clinical trials for i-ID. The performance and reliability of i-IDs are typically evaluated at the regulatory approval stage, while their impact on patient outcomes is assessed post-launch. The utility of i-IDs is often expressed through qualitative evidence based on surgical use. Thus, a framework for evaluating the clinical utilities and issues of i-ID in clinical trials is essential. The themes identified in this study can serve as evaluation criteria in future clinical trials of i-IDs involving human factors, such as 1) alteration of decision-making, 2) utility for microsurgical procedures, and 3) troubles or surgical interruptions.

As i-ID leveraging innovative technologies may yield remarkable patient outcomes or unexpected risks, early evaluation post-launch is critical. However, synthesising clinical evidence from clinical trials is time-intensive because quantitative SRs require substantial data for statistical analysis. Our findings suggest that the QES method could be a powerful tool for evaluating i-ID even with limited clinical data.

This study has some limitations. First, the clinical studies on iOCT included in this analysis primarily involve low-level evidence (levels 3–4), such as case series reports. Applying QES to datasets that include future randomised controlled trial results could strengthen the credibility of the evidence. Second, this study focused exclusively on iOCT. To demonstrate the versatility of QES in qualitative evidence aggregation, additional case studies involving other intraoperative modalities, such as iMRI and iCT, are needed. These modalities also influence intraoperative decision-making. Third, as with all QES studies, there is an inherent risk of bias due to human involvement in the analysis process. To mitigate this, we adopted the reviewers’ familiarisation and independent review processes in accordance with established QES methodologies. Machine learning-assisted synthesis is a potential approach for objectively and automatically analysing evidence without human bias [38, 39]. However, automatic analysis based on machine learning and natural language processing with “black box” algorithms may carry the risk of incorrect conclusions and lack reliability in analysis for small datasets; therefore, benchmarking against human analysis results is generally performed [40]. The manual synthesis outcomes from this study provide a foundation for future automated approaches.

## Conclusions

This study demonstrated the feasibility of using QES to systematically extract and synthesise both the clinical utilities and issues of i-ID from clinical reports, with iOCT serving as a case study. By employing QES, we provided a comprehensive understanding of the benefits and challenges associated with iOCT. Our findings underscore iOCT’s significant role in enhancing intraoperative decision-making, specifically in tasks such as membrane peeling and in detecting conditions such as full-thickness macular holes that were not identified preoperatively. Additionally, this study identified critical technical challenges associated with iOCT, including device malfunctions and procedural interruptions. These insights are vital for guiding improvements in device design and their integration into surgical workflows.

The application of QES facilitated a thorough investigation into the clinical utilities and issues of iOCT, encouraging the application of this method in the ongoing evaluation of i-ID technologies. This initial experience with QES confirms its potential for synthesising qualitative clinical data and suggests its applicability beyond iOCT to other i-ID modalities. Providing comprehensive information to stakeholders about the advantages and disadvantages of innovative i-IDs facilitates safer therapy and their implementation in operating rooms. Furthermore, as intraoperative innovations involving human factors expand into new clinical domains such as precision medicine [41], surgical robots [42], and intraoperative diagnostics [43], the use of QES, as demonstrated in this study, will offer a timely and effective approach for synthesising evidence. By systematically addressing both utilities and technical issues, this method enhances the reliability of findings and provides a solid foundation for decision-making by policymakers and medical specialists.

## Supplementary Information

Supplementary Material 1.

## Data Availability

The authors declare that all the data included in this study are available within the paper.

## Abbreviations

SR: Systematic Review
i-ID: Intraoperative Imaging Devices
iOCT: Intraoperative Optical Coherence Tomography
DISCOVER: Determination of feasibility of Intraoperative Spectral domain microscope Combined/integrated OCT Visualisation during En face Retinal and ophthalmic surgery
PIONEER: Prospective Intraoperative and Perioperative Ophthalmic ImagiNg with Optical CoherEncE TomogRaphy
QoL: Quality of Life
Qual-SR: Qualitative Systematic Review
PRISMA: Preferred Reporting Items for Systematic reviews and Meta-Analyses
QDA: Qualitative Data Analysis
